# *Primula daguanensis* (Primulaceae), a new species from Yunnan, China

**DOI:** 10.3897/phytokeys.270.171019

**Published:** 2026-02-03

**Authors:** Jian Ru, Xin-Yu Cheng, Bo Xu, Lian-Jin Guo, Wen-Bin Ju

**Affiliations:** 1 Key Laboratory for Regional Plants Conservation and Ecological Restoration of Northeast Jiangxi, College of Life Science, Shangrao Normal University, Shangrao, 334001, Jiangxi, China College of Life Science, Shangrao Normal University Shangrao China; 2 Mountain Ecological Restoration and Biodiversity Conservation Key Laboratory of Sichuan Province, Chengdu Institute of Biology, Chinese Academy of Sciences, Chengdu, 610041, Sichuan, China Chengdu Institute of Biology, Chinese Academy of Sciences Chengdu China

**Keywords:** New species, phylogeny, *Primula daguanensis*, taxonomy

## Abstract

*Primula daguanensis* W.B.Ju, B.Xu & J.Ru is described and illustrated as a new species from Daguan County, Yunnan Province, China. It is assigned to the *Primula* sect. *Petiolares* subsect. *Davidii* based on its conspicuous bud scales, which are membranous and persistent at anthesis, and its coriaceous, efarinose leaves. Molecular phylogenetic analysis based on the nuclear ribosomal internal transcribed spacer (nrITS) shows it to be closely related to *P. tridentifera* of subsect. *Petiolares*, and morphological comparisons support its recognition as a distinct new species. It resembles *P. epilosa*, *P. tridentifera*, and *P. bergenioides* but can be distinguished by the following characters: petiole 1.0–4.5 cm long; leaves efarinose and obovate to oblanceolate, with undulate-dentate margins; abaxial reticulate veins inconspicuous, lateral veins 5–7 pairs; calyx parted to one-third, with broadly triangular lobes; and corolla lobes truncate at the apex, dentate, and bearing an annulus. *P. daguanensis* is currently known only from the collection locality, and its conservation status is assessed as Data Deficient (DD) according to the IUCN Red List Categories and Criteria.

## Introduction

*Primula* Linnaeus (Linnaeus 1753) is one of the larger genera in Primulaceae, comprising more than 500 species (POWO 2025). The genus is primarily distributed in the temperate and alpine regions of the Northern Hemisphere, with only a very few species occurring in the Southern Hemisphere (Hu 1990a; Hu and Kelso 1996; Richards 2003). More than 300 species of *Primula* are native to China, mainly concentrated in the southwestern regions (Hu 1990a).

*Primula* sect. *Petiolares* was established by Pax in 1889 (Pax 1889) and includes approximately 70 species. Its center of distribution lies in the Himalaya–Hengduan Mountains, with some species extending to Kashmir, central China, and other regions (Hu 1994). Members of this section typically bear scales that are persistent at the base of the leaf rosettes or deciduous before anthesis. The capsule is globose and nearly membranous, dehiscing irregularly at maturity (Hu 1990a). Following Smith and Fletcher (1944), sect. *Petiolares* is subdivided into five subsections: *Chartacea* Smith & Forrest, *Davidii* Smith & Forrest, *Griffithii* Smith & Forrest, *Petiolaris-Sonchifolia* Smith & Forrest, and *Tongolensis* Smith & Forrest.

In recent years, extensive fieldwork and taxonomic studies have led to the discovery of numerous new species within this section, including *Primula bergenioides* C.M.Hu & Y.Y.Geng (Hu and Geng 2003), *P. tenuituba* C.M.Hu & Y.Y.Geng (Hu and Geng 2003), *P. dejuniana* G.Hao, C.M.Hu & Y.Xu (Xu et al. 2014), *P. wawushanica* G.Hao, C.M.Hu & Y.Xu (Xu et al. 2016), *P. chimingiana* G.Hao, S.Yuan & D.X.Zhang (Yuan et al. 2018), *P. luteoflora* X.F.Gao & W.B.Ju (Ju et al. 2018), *P. surculosa* Y.Xu & G.Hao (Xu et al. 2022), *P. jiaozishanensis* Z.K.Wu, W.H.Yang & Y.Wu (Wu et al. 2023), *P. pingbaensis* N.Zhang, X.Q.Jiang & Z.K.Wu (Zhang et al. 2023), and *P. meishanensis* K.Huang & Z.X.Fu (Li et al. 2024).

During botanical fieldwork in Daguan County, Yunnan Province, China, an unidentified population of *Primula* was discovered, photographed in situ, and collected for morphological and molecular analyses. Comparisons with closely related and morphologically similar species support recognition of this population as a new species in *Primula* sect. *Petiolares* subsect. *Petiolares*. A detailed description is provided here based on field observations of living plants and examination of herbarium specimens.

## Materials and methods

### Morphological observation

The specimen was collected from Daguan County, Yunnan Province. Habitat, growth habit, and phenology were recorded during botanical fieldwork. Morphological observations, measurements, and the new species description were based on living plants observed in the field and herbarium specimens. Comparison of morphological characters between the new species and its allies was conducted based on specimens obtained from their respective type localities, online specimen images from the Chinese Virtual Herbarium (https://www.cvh.ac.cn/) and JSTOR Global Plants (https://plants.jstor.org/), as well as relevant literature (Hu 1990b; Hu and Geng 2003; Hu and Kelso 1996). The conservation status was assessed following the IUCN Red List Categories and Criteria (IUCN Standards and Petitions Committee 2024).

### Phylogenetic study

To determine the phylogenetic position of *P. daguanensis* within *Primula*, we performed phylogenetic analysis based on nrITS sequences from 46 species of *Primula*, including *P. daguanensis*, and used *Androsace maxima* L. and *Lysimachia chapaensis* Merr. as outgroups. The nrITS sequences of *P. bergenioides*, *P. daguanensis*, *P. epilosa*, and *P. tridentifera* were newly generated in this study, whereas those of the remaining species were retrieved from GenBank. The GenBank accession numbers are provided in Suppl. material 1.

Genomic DNA was extracted from silica-gel-dried leaves using a CTAB protocol. Sequencing libraries were prepared using the BGI Optimal DNA Library Prep Kit (BGI, Shenzhen, China). Sequencing was performed on a DNBSEQ-T7 platform (BGI, Shenzhen, China) to generate paired-end 150 bp reads. Raw reads were filtered using SOAPnuke v2.2.6 (Chen et al. 2018) with the following parameters: SOAPnuke filter -n 0.001 -l 10 -q 0.5 -Q 2, and clean reads were used for nrITS assembly and subsequent analyses.

The nrITS sequences were assembled from the filtered reads using GetOrganelle v1.7.7.1 (Jin et al. 2020). The ITS1, 5.8S, and ITS2 regions were extracted, aligned using the MAFFT plugin in Geneious v2024.0.5 (https://www.geneious.com), and trimmed. Maximum likelihood (ML) phylogenetic inference was performed in IQ-TREE v2.3.6 (Minh et al. 2020) using the ITS alignment. The best-fit nucleotide substitution model was selected according to the Bayesian Information Criterion (BIC), and SYM+I+G4 was chosen. Node support was assessed with 1,000 ultrafast bootstrap replicates.

## Results

**Figure 1.**
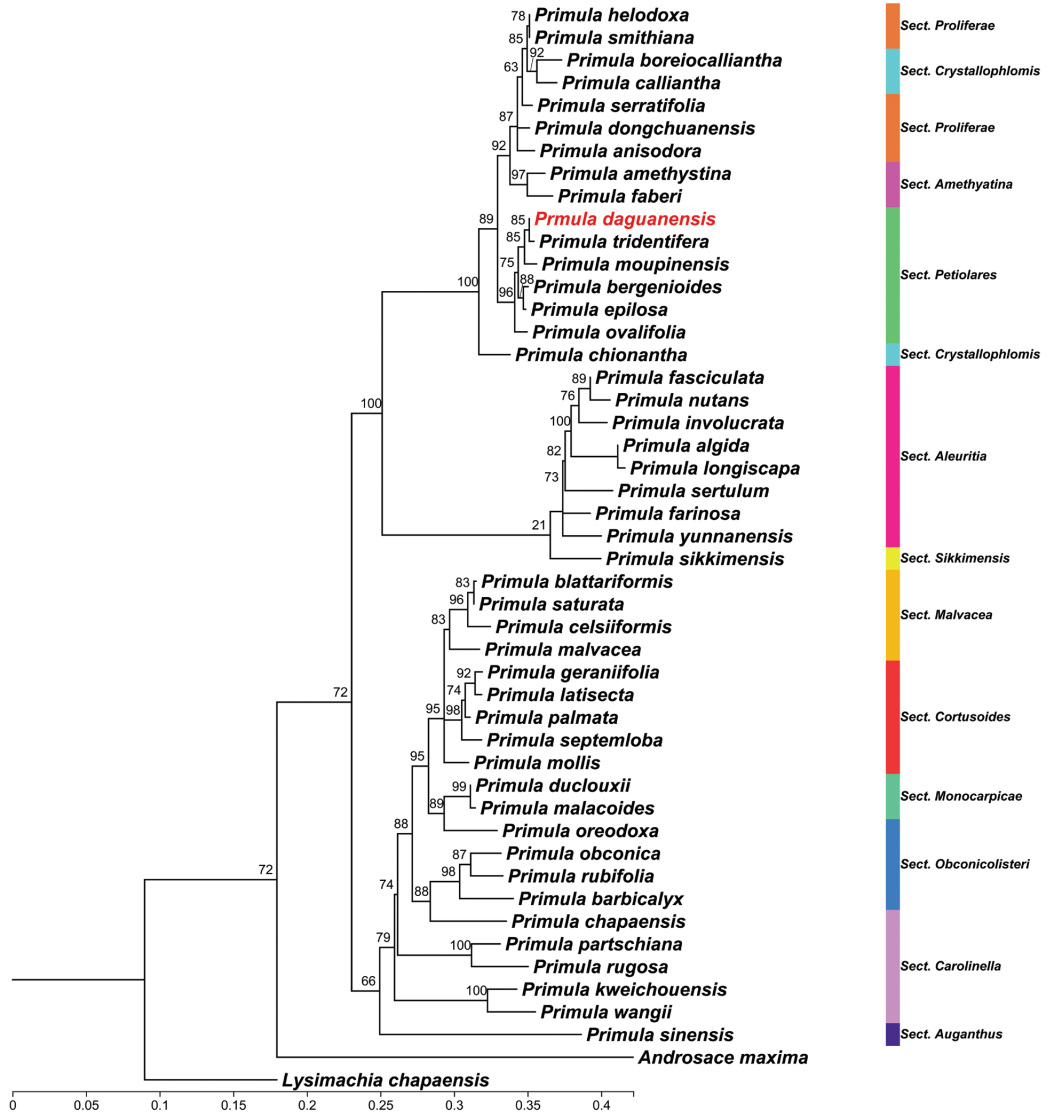
Maximum likelihood phylogenetic tree of *Primula* based on nrITS sequence data. Ultrafast bootstrap support values are shown above branches.

The phylogenetic tree inferred from nrITS sequences (Fig. 1) recovers *P. daguanensis* in a strongly supported clade with six species of section *Petiolares* (BS = 96%). Within this clade, *P. daguanensis* is sister to *P. tridentifera* with moderate support (BS = 85%).

### Taxonomic treatment

#### Primula daguanensis

Taxon classificationPlantaeEricalesPrimulaceae

W.B.Ju, B.Xu & J.Ru sp. nov.

6EB2F5A9-ADCD-5035-855C-344A38F57457

urn:lsid:ipni.org:names:77376395-1

Figs 4, 5

##### Type.

**Figure 2.**
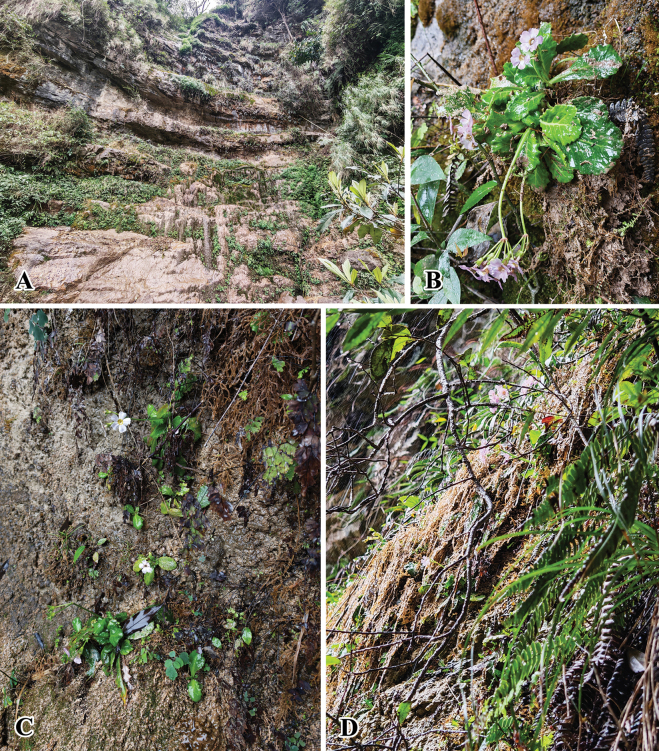
Habitat of *Primula daguanensis* sp. nov. (**A–D**). Photographed by W.B.Ju.

China • Yunnan: Daguan County, growing on limestone cliff with running water (Fig. 2). 27°43'48.57"N, 103°53'01.21"E, elevation ca. 1136 m, 30 March 2025, *W.B.Ju J01397* (holotype: CDBI! Barcode number: CDBI0298288; isotypes: KUN!, PE!)

##### Diagnosis.

**Figure 3.**
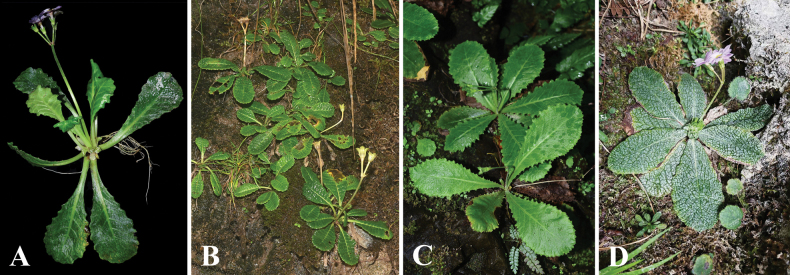
*Primula daguanensis* and three allied species. **A**. *P. daguanensis*. **B**. *P. epilosa*. **C**. *P. bergenioides*. **D**. *P. tridentifera*. Photographed by W.B.Ju.

*Primula daguanensis* is morphologically similar to *P. epilosa*, *P. tridentifera*, and *P. bergenioides* in having a stout rhizome, membranous bud scales persistent at thesis, and a similar leaf outline (Fig. 3), but differs by a combination of characters: leaves efarinose, with undulate-dentate margins; adaxial surface smooth; abaxial reticulate veins inconspicuous; lateral veins 5–7 pairs; petiole 1.0–4.5 cm long; calyx parted to one-third of its length, with broadly triangular lobes; corolla bears an annulus, with lobes truncate at apex and dentate.

##### Description.

Perennial herb, rhizome short and stout, with numerous fibrous roots. Membranous scales present at the base of leaves, oblong, 1.5–2.5 cm long, with glandular hairs along the margin and a foliaceous apex with dentate margins. Young leaves are not yet fully expanded at anthesis. Leaf blades obovate to oblanceolate, 4.5–8.8 cm long, 3.0–4.5 cm wide, base gradually attenuate and cuneate, decurrent into a winged petiole 1.0–4.5 cm long; margins undulate-dentate. Leaves coriaceous, efarinose, adaxially smooth; abaxially with glandular hairs along the veins and inconspicuous reticulate venation. Midrib relatively broad, flat adaxially and raised abaxially; lateral veins 5–7 pairs. Scapes 7–12 cm tall, glandular-pubescent; umbel with (2) 4–7 flowers; bracts ovate-triangular, apex gradually acuminate, ca. 4 mm long, margins with glandular hairs. Pedicels ca. 1.8 cm long, glandular-pubescent. Flowers heterostylous. Calyx campanulate, ca. 0.8 cm long, glandular-pubescent, parted to one-third of its length; lobes broadly triangular, apex acute. Corolla purplish-red, ca. 2.3 cm in diameter; with an annulus at the throat; lobes obovate, ca. 7.5 mm wide, apex truncate with dentate. Pin flowers: corolla tube ca. 11 mm long; stamens inserted ca. 6 mm above the base of the tube; style ca. 8 mm long, slightly exceeding the corolla tube. Thrum flowers: corolla tube ca. 14 mm long; stamens inserted ca. 11 mm above the base of the tube; style ca. 4 mm long.

**Figure 4.**
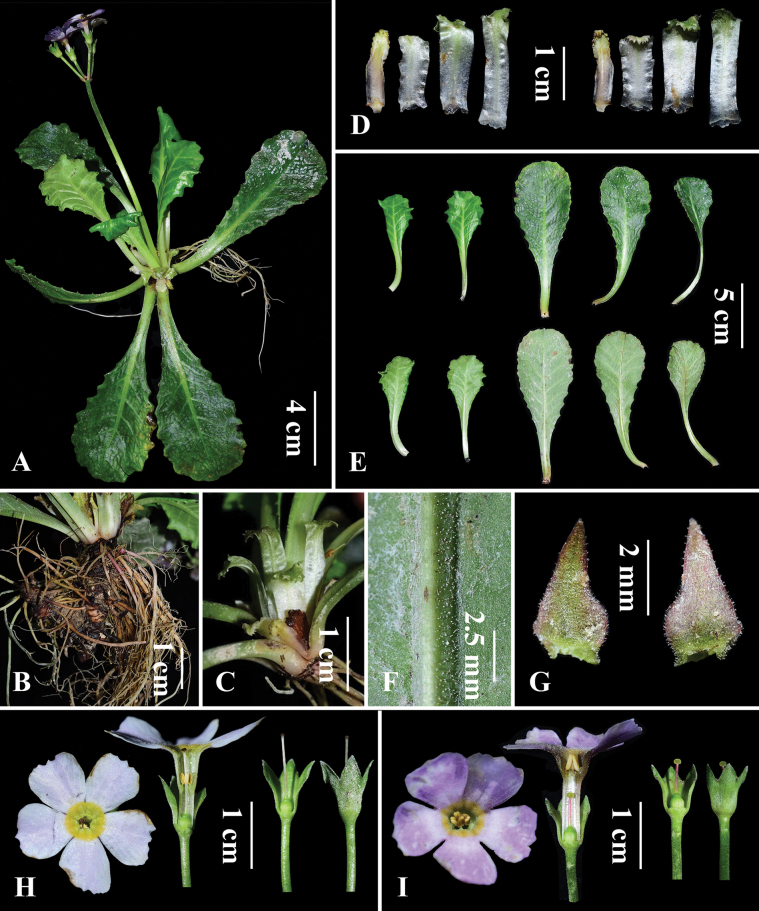
*Primula daguanensis* sp. nov. **A**. Plant. **B**. Rhizome. **C, D**. Scales. **E**. Leaves. **F**. Abaxial leaf surface showing glandular hairs on the midrib. **G**. Bract. **H**. Pin flowers. **I**. Thrum flower. Photographed by W.B.Ju.

**Figure 5.**
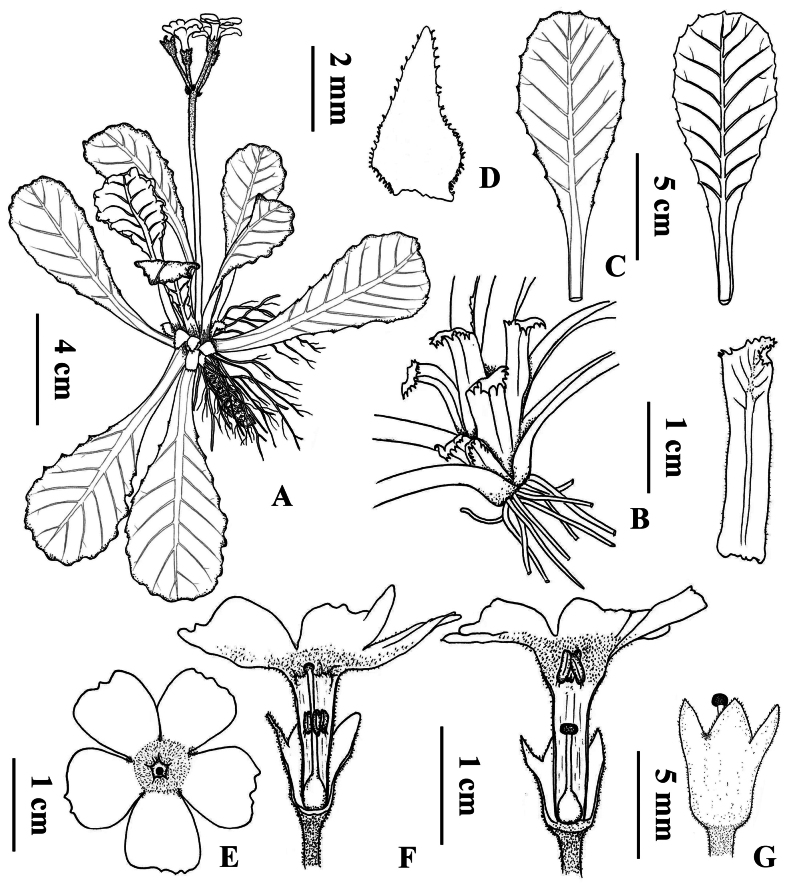
*Primula daguanensis* sp. nov. **A**. Plant. **B**. Scales. **C**. Leaves. **D**. Bract. **E**. Top view of the flower. **F**. Pin flowers (left) and thrum flower (right). **G**. Calyx of thrum flower. Drawn by Dr. Zhen-long Liang.

##### Etymology.

The specific epithet refers to Daguan County, Yunnan Province, where the new species was discovered and collected.

##### Vernacular name.

Simplified Chinese: 大关报春; Chinese pinyin: Dà guān bào chūn.

##### Phenology.

Flowering in March; fruiting from April to May.

##### Distribution and habitat.

*Primula daguanensis* is currently known only from the collection locality, Cuihua Town, Daguan County, Yunnan Province, where it grows on limestone cliffs with running water at an elevation of 1136 m (Fig. 2).

##### Preliminary conservation assessment.

Based on current botanical fieldwork, *Primula daguanensis* is known only from Cuihua Town, Daguan County, Yunnan Province, and exhibits a highly restricted distribution range. According to the IUCN Red List Categories and Criteria (IUCN Standards and Petitions Committee 2024), it is provisionally assessed as Data Deficient (DD) due to insufficient data. Further botanical surveys in adjacent areas are required to better evaluate its distribution and conservation status.

## Discussion

*Primula* sect. *Petiolares* subsect. *Davidii* is characterized by lax, membranous bud scales; the presence of farinipotent hairs without visible farina; coriaceous leaves that often persist through winter into the following spring; frequently bullate adaxial surfaces; and abaxial surfaces bearing honeycomb-like reticulate venation, often with hairs along the veins. The species is primarily distributed in western China (Smith and Fletcher 1944).

The new species *P. daguanensis* shares several morphological characteristics with members of subsect. *Petiolares*. It is most similar to *P. epilosa*, *P. tridentifera*, and *P. bergenioides*. Although only six of ca. 70 species of sect. *Petiolares* have been analyzed; our phylogenetic results place *P. daguanensis*, *P. tridentifera*, *P. bergenioides*, and *P. epilosa* within a strongly supported sect. *Petiolares* clade together with *P. moupinensis* and *P. ovalifolia* (BS = 96%; Fig. 1). *Primula moupinensis* is recovered as sister to the *P. daguanensis* and *P. tridentifera* clade (BS = 85%; Fig. 1), to the exclusion of the other morphologically similar taxa, thus rendering subsect. *Petiolares* paraphyletic. Since this finding is based only on sparse taxon sampling and a single-locus nrITS phylogeny, it should be further evaluated with denser sampling and additional markers. *Primula daguanensis* can be readily distinguished by a distinct combination of characters, including petiole length, leaf margin morphology, leaf surface sculpturing, as well as calyx and corolla features. *Primula breviscapa* (also subsect. *Petiolares* but not analyzed here) is also reported from Daguan; however, it differs markedly from *P. daguanensis* in having membranous leaves tapering into a long lanuginose petiole, closely eroso-dentate margins, and flowers often appearing before the leaves.

The petiole of *P. daguanensis* is 1.0–4.5 cm long, which is significantly longer than that of *P. epilosa* (0.5–2.5 cm), and contrasts with the nearly sessile leaves of *P. tridentifera* and *P. bergenioides*. Its leaf blades are obovate to oblanceolate, with undulate-dentate margins; the adaxial surface is smooth, and the abaxial reticulate venation is inconspicuous. Furthermore, the leaves are entirely efarinose. In contrast, *P. epilosa* and *P. tridentifera* have farinose abaxial surfaces, bullate adaxial surfaces, and prominently raised reticulate veins abaxially.

The calyx of *P. daguanensis* is parted to one-third of its length, with broadly triangular, acute lobes. In comparison, the other species differ in the depth of calyx division and lobe shape, particularly *P. tridentifera*, which is notable for its three-toothed calyx lobes. The corolla of *P. daguanensis* is further distinguished by its dentate lobes and the presence of an annulus at the throat, both features absent in *P. epilosa* and *P. bergenioides*. A detailed comparison of the morphological differences between *P. daguanensis* and these species is provided in Table 1.

**Table 1.** Morphological comparison of *Primula daguanensis*, *P. epilosa*, *P. tridentifera*, and *P. bergenioides*.

Characters	*Primula daguanensis*	*P. epilosa*	*P. tridentifera*	*P. bergenioides*
**Petiole**	1.0–4.5 cm	0.5–2.5 cm	extremely short and indistinct	almost obsolete
**Leaf blade**	obovate to oblanceolate	elliptic-obovate to obovate-oblanceolate	obovate to obovate-oblong	oblong-elliptic to elliptic-obovate
	margin undulate-dentate	margin hydathode-dentate	margin lobulate-dentate	margin irregularly crenate-dentate
	adaxially smooth, abaxially reticulate veins inconspicuous	adaxially bullate, with reticulate veins prominently raised on the abaxial surface	adaxially bullate, with reticulate veins prominently raised on the abaxial surface	both surfaces smooth, with reticulate veins inconspicuous
	efarinose	abaxially white farinose at maturity	abaxially densely white farinose, adaxially covered with white farina or glandular hairs	efarinose
**Calyx**	parted to 1/3; lobes broadly triangular, glandular, apex acute	parted to middle; lobes ovate to ovate–lanceolate, ciliolate, apex acute to short acuminate	parted to ca. 1/3; lobes ovate to ovate–oblong, margin 3-toothed at apex	parted to 1/3; lobes ovate, apex acute
**Corolla**	lobes dentate at apex	lobes emarginate or 2-cleft at apex	lobes 2-cleft at apex	lobes emarginate at apex
	annulate	exannulate	—	exannulate

## Supplementary Material

XML Treatment for Primula daguanensis

## Additional information

### Use of AI

No use of AI was reported.

### Author ORCIDs

Jian Ru https://orcid.org/0000-0003-1268-3243

Xin-Yu Cheng https://orcid.org/0009-0005-8920-2112

Bo Xu https://orcid.org/0000-0003-3507-9321

Lian-Jin Guo https://orcid.org/0000-0001-9078-1311

Wen-Bin Ju https://orcid.org/0000-0002-5665-6290

### Data availability

All of the data that support the findings of this study are available in the main text or Supplementary Information.

## Supplementary materials

Supplementary material 1 Taxa used in the phylogenetic analyses and their GenBank accession numbers Data type xlsx https://binary.pensoft.net/file/1526823This dataset is made available under the Open Database License (http://opendatacommons.org/licenses/odbl/1.0/). The Open Database License (ODbL) is a license agreement intended to allow users to freely share, modify, and use this Dataset while maintaining this same freedom for others, provided that the original source and author(s) are credited. Jian Ru, Xin-Yu Cheng, Bo Xu, Lian-Jin Guo, Wen-Bin Ju
